# DNA metabarcode analyses reveal similarities and differences in plant microbiomes of industrial hemp and medicinal *Cannabis* in China

**DOI:** 10.3389/fmicb.2025.1524703

**Published:** 2025-04-15

**Authors:** Jiayang Li, Hong Zhang, Songhua Long, Wenting Li, Tuhong Wang, Jian Yu, Ying Zhou, Shuo Zou, Hongjian Zhu, Jianping Xu, Yi Cheng

**Affiliations:** ^1^Hunan Provincial Key Laboratory for Biology and Control of Plant Diseases and Insect Pests, Hunan Agricultural University, Changsha, Hunan, China; ^2^Institute of Bast Fiber Crops and Center of Southern Economic Crops, Chinese Academy of Agricultural Sciences, Changsha, Hunan, China; ^3^Shenzhen Noposion Crop Science Co., Ltd., Shenzhen, Guangdong, China; ^4^Xishuangbanna Dai Autonomous Prefecture Tea Industry Development Service Center, Jinghong, Yunnan, China; ^5^Institute of Agricultural Sciences of Xishuangbanna Prefecture of Yunnan Province, Jinghong, Yunnan, China; ^6^Changsha Agricultural and Rural Bureau, Changsha, Hunan, China; ^7^Department of Biology, McMaster University, Hamilton, ON, Canada; ^8^Hunan Provincial Key Laboratory of the Traditional Chinese Medicine Agricultural Biogenomics, Changsha Medical University, Changsha, China

**Keywords:** bacterial diversity, community composition, *Cannabis sativa* L., community differences, functional prediction

## Abstract

Endophytic bacteria within plant tissues play crucial roles in plant health, stress tolerance, and contribute to the metabolite diversity of host plants. *Cannabis sativa* L. is an economically significant plant, with industrial hemp (IH) and medicinal *Cannabis* (MC) being the two main cultivars. However, the composition and functional traits of their endophytic bacterial communities in roots and leaves are not well understood. In this study, DNA metabarcode sequencing were employed to compare the bacterial communities between IH and MC. Significant differences were observed in the root and leaf niches. IH roots were enriched with stress-tolerant bacteria, while MC roots showed higher levels of biofilm-forming bacteria. In leaves, differences were even more pronounced, particularly in the abundance of Gram-negative bacteria, potential pathogens, stress-tolerant bacteria, and biofilm-forming bacteria. PICRUSt2 functional predictions revealed differences in nitrogen metabolism and secondary metabolite biosynthesis pathways in different cultivars and niches, while FAPROTAX analysis highlighted variations in carbon, nitrogen, and sulfur cycling functions. These findings underscore the distinct roles of bacterial communities in regulating plant health, stress responses, and metabolic processes in different niches and cultivars, providing insights for improving cultivation practices and plant resilience.

## 1 Introduction

*Cannabis sativa* L. is a versatile crop widely utilized in fiber production (Panthapulakkal and Sain, 2007), pharmaceutical development (Atakan, 2012), and construction materials (Zimniewska, 2022). Cultivars of this species are commonly divided into two types, industrial hemp (IH) and medical *Cannabis* (MC). IH is primarily grown for its high yield of fiber and seed (Ahmed et al., 2022), while MC is distinguished by its active compounds, particularly cannabidiol (CBD), making it highly valuable in the medical (Crescente et al., 2023; Song et al., 2023) and recreational fields. Recent advances in the biological study of *C. sativa* L. and its symbiotic microorganisms (Islam et al., 2023; Law et al., 2020) have underscored the importance of endophytic bacteria in promoting plant growth, enhancing disease resistance, and augmenting environmental adaptability (Zhuang et al., 2021).

Endophytic bacteria are microbial communities that colonize the internal tissues (Guzmán-Guzmán et al., 2023) of plants without apparent disease symptoms (Lundberg et al., 2012). Endophytic bacteria engage in intricate symbiotic interactions with the host plants, where they contribute to plant health through multiple mechanisms. These include directly facilitating nutrient uptake by promoting nitrogen fixation and phosphate solubilization, as well as indirectly enhancing plant immunity (Xiong et al., 2024) and improving resilience to environmental stressors such as drought and pathogens (Balthazar et al., 2022). *Pseudomonas* in *C. sativa* L. have been reported to promote plant growth via phytohormone production and biocontrol activity. Additionally, these bacteria may influence the biosynthesis of key secondary metabolites such as tetrahydrocannabinol (THC) and cannabidiol (CBD) by modulating plant metabolic pathways (Balthazar et al., 2022).

The composition of endophytic bacteria is largely shape by host genotype (Liu et al., 2022; Wang Y.C. et al., 2022), environmental conditions (Santoyo, 2022), and tissue-specific niches (Zhang et al., 2022). Roots and leaves support distinct microbial assemblages due to differences in nutrient availability (Francioli et al., 2018; Potter et al., 2023), plant defense strategies (Lu et al., 2025), and microbial colonization routes (Zeng et al., 2023). Root-associated endophytes are primarily influenced by rhizodeposition (Xu et al., 2020), a process in which plant roots exude organic acids, flavonoids, and terpenoids that selectively recruit beneficial bacteria. For instance, *Pseudomonas* (Orozco-Mosqueda et al., 2018) enhances plant health through antimicrobial compound production, *Bacillus* (Igiehon and Babalola, 2018) promotes growth via phytohormone synthesis, and *Rhizobium* (Xiao et al., 2017) facilitates nitrogen fixation. In contrast, leaf endophytes are shaped by phyllosphere chemistry and environmental exposure (Chen et al., 2020). Microbial recruitment occurs through airborne deposition, insect-mediated transmission (Li et al., 2022), and cuticle adhesion (Sohrabi et al., 2023). Adaptations such as UV resistance and biofilm formation enable these microbes to persist in dynamic aerial environments. Despite growing interest, research on the composition and functional roles of endophytic bacterial communities in *C. sativa* L. remain limited, necessitating further studies to unravel the underlying mechanisms shaping these microbial communities (Chen et al., 2024; Xiao et al., 2023).

In *Cannabis*, several studies have explored the composition and ecological functions of associated microbial communities. Research has shown that bacterial communities colonizing the roots of industrial hemp contribute significantly to plant growth promotion (Taghinasab and Jabaji, 2020; Wei et al., 2021). Endophytic microorganisms can enhance plant growth by synthesizing phytohormones such as indole-3-acetic acid (IAA) (Etesami and Glick, 2024), facilitating nitrogen fixation (Yan et al., 2024), solubilizing phosphate (Chen et al., 2021), and producing siderophores (Schalk, 2025). In addition to promoting plant growth, these microbes enhance abiotic stress tolerance by producing osmoprotectants (e.g., proline, trehalose) (Anand et al., 2023), antioxidants (e.g., catalase, superoxide dismutase) (Hwang et al., 2022), and modulating stress-responsive gene expression (Jan et al., 2024). Furthermore, root- and leaf-associated microbiomes contribute to disease resistance via multiple strategies, including the synthesis of antifungal and antibacterial metabolites, the induction of systemic resistance (ISR) (Watts et al., 2023), and the priming of plant immune responses (Pal et al., 2022).

The metabolomic and physiological differences among *Cannabis* varieties play a crucial role in shaping their associated microbial communities (Lobato et al., 2024). The production of cannabinoids, terpenoids, and flavonoids can exert selective pressures on bacterial colonization and community composition. High-THC cultivars, for example, have been shown to host distinct microbial communities compared to fiber-type hemp, suggesting that secondary metabolites can influence microbial assembly (Ahmad et al., 2024). These findings highlight the complex interplay between *Cannabis* secondary metabolism and microbial ecology, with potential implications for optimizing plant health and productivity.

This study aims to explore the differences in the composition and function of endophytic bacterial communities between IH and MC, with the ultimate goal of potentially enhancing their respective desired phenotypes by manipulating their endophytic bacteria. The specific objectives are: (1) to compare the bacterial communities present in the roots and leaves of both industrial hemp and medicinal *Cannabis* through DNA metabarcode sequencing; (2) to identify bacterial taxa that show differential abundance between the two types, which could serve as potential biomarkers; and (3) to perform a predictive functional analysis using PICRUSt2 and FAPROTAX to reveal functional distinctions within the bacterial communities associated with each cultivar type. This study advances the understanding of plant-microbe interactions by revealing distinct microbial signatures in industrial hemp and medicinal *Cannabis*, emphasizing their ecological and functional significance. The findings lay a foundation for microbiome-based crop improvement strategies, including enhancing plant resilience, optimizing growth conditions, and implementing microbiome-informed cultivation practices tailored for industrial and medicinal applications.

## 2 Materials and methods

### 2.1 Sample collection

In this study, all root and leaf samples (root samples: 112, leaf samples: 79) were collected from eight locations across six provinces in China where *Cannabis* is cultivated (Table 1). All sampling was conducted during the flowering period of the hemp plants. Field samples were transported to the laboratory on dry ice to preserve their integrity. Upon arrival, the samples were rinsed 3–4 times with sterile water until the roots were clean, and the leaf surfaces were free of soil. The cleaned samples underwent surface sterilization by sequential immersion in 75% ethanol for 30 s, 1% sodium hypochlorite solution for 4 min, and 95% ethanol for 10 s, followed by three rinses with sterile distilled water (Li et al., 2023; Misaghi and Donndelinger, 1990). After sterilization, the samples were cut into small pieces and stored at −80°C for subsequent microbial DNA extraction.

**Table 1:** Sample information.

Province	Region	Latitude/Longitude	Cultivar type
Heilongjiang	Harbin	126.44/45.59	Medical *Cannabis*
			Industrial hemp
	Daqing	125.23/46.67	Industrial hemp
Jilin	Changchun	125.09/43.72	Industrial hemp
			Industrial hemp
Hunan	Yuanjiang	112.36/28.76	Industrial hemp
			Medical *Cannabis*
			Medical *Cannabis*
Yunnan	Chuxiong	101.55/25.14	Medical *Cannabis*
	Qujing	103.75/25.85	Medical *Cannabis*
Shandong	Tai’an	116.09/35.97	Industrial hemp
Anhui	Lu’an	116.52/31.81	Industrial hemp

### 2.2 DNA extraction, sequencing, and processing

The total genomic DNA of each endophytic microbial community was extracted following the protocol by the E.Z.N.A.® Soil DNA Kit (Omega Bio-tek, Norcross, GA, United States). The extracted DNA’s quality and quantity were evaluated by 1% agarose gel electrophoresis and a NanoDrop 2000 spectrophotometer (Thermo Scientific, United States). The primers 799F (5′-AACMGGATTAGATAC CCKG-3′) and 1392R (5′-ACGGGCGGTGTGTRC-3′) were selected for the first round of PCR amplification of the V5–V7 variable region, and the primers 799F (5′-AACMGGATTAGATACCCKG-3′) and 1193R (5′-ACGTCATCCCCACCTTCC-3′) were selected for the second round of PCR amplification of the V5–V7 variable region. Library construction of the purified PCR products was performed using the NEXTFLEX Rapid DNA-Seq Kit, following these steps: (1) adapter ligation, (2) bead-based selection to remove self-ligated adapter fragments, (3) PCR amplification to enrich the library template, and (4) bead-based purification to obtain the final library. High-throughput sequencing was then conducted on the Illumina NextSeq 2000 platform (Shanghai Majorbio Bio-Pharm Technology Co., Ltd.) (Bouchez et al., 2016). The raw sequencing data have been submitted to the NCBI Sequence Read Archive (Re: PRJNA1169634, Le: PRJNA1172293).

### 2.3 Bioinformatics analysis

The raw reads were quality-controlled using fastp (version 0.19.6) by trimming low-quality bases (Q < 20) with a sliding window of 50 bp and removing reads shorter than 50 bp or containing N bases. The processed paired-end reads were then merged using FLASH software (version 1.2.11) with a minimum overlap length of 10 bp and a maximum mismatch ratio of 0.2. The DADA2 plugin within the Qiime2 pipeline was employed to denoise the quality-controlled and merged sequences. Sequences identified as chloroplast or mitochondrial were excluded from all samples. To minimize the influence of sequencing depth on subsequent Alpha and Beta diversity analyses, the sequencing depth was rarefied to maintain an average sequence coverage of 98.00% for each sample. Taxonomic classification of OTUs was carried out using the Naive Bayes classifier, based on the Silva 16S rRNA gene database (v. 138) within Qiime2 (Edgar, 2013).

### 2.4 Statistical analyses

All statistical analysis was conducted using the Shanghai MajorBio Cloud Platform.^1^ The functional potential of the microbial communities was predicted using PICRUSt2 and FAPROTAX. For diversity analysis, alpha diversity indices (Sobs, Shannon, ACE) were calculated using Mothur (version 1.30.1), and beta diversity was assessed using the Bray-Curtis distance metric, followed by PCoA analysis in QIIME (version 1.17). Significant differences in community composition between groups were evaluated using the ANOSIM group difference test. To identify significant differences in dominant bacterial genera between groups, the Wilcoxon rank-sum test was applied. Additionally, LEfSe (LDA score > 3.5) was used to identify biomarker taxa differentiating the groups. Source tracking analysis was conducted using FEAST (Shenhav et al., 2019).

## 3 Results

### 3.1 Endophytic bacterial differences within and between medicinal *Cannabis* and industrial hemp from Harbin

**Figure 1:**
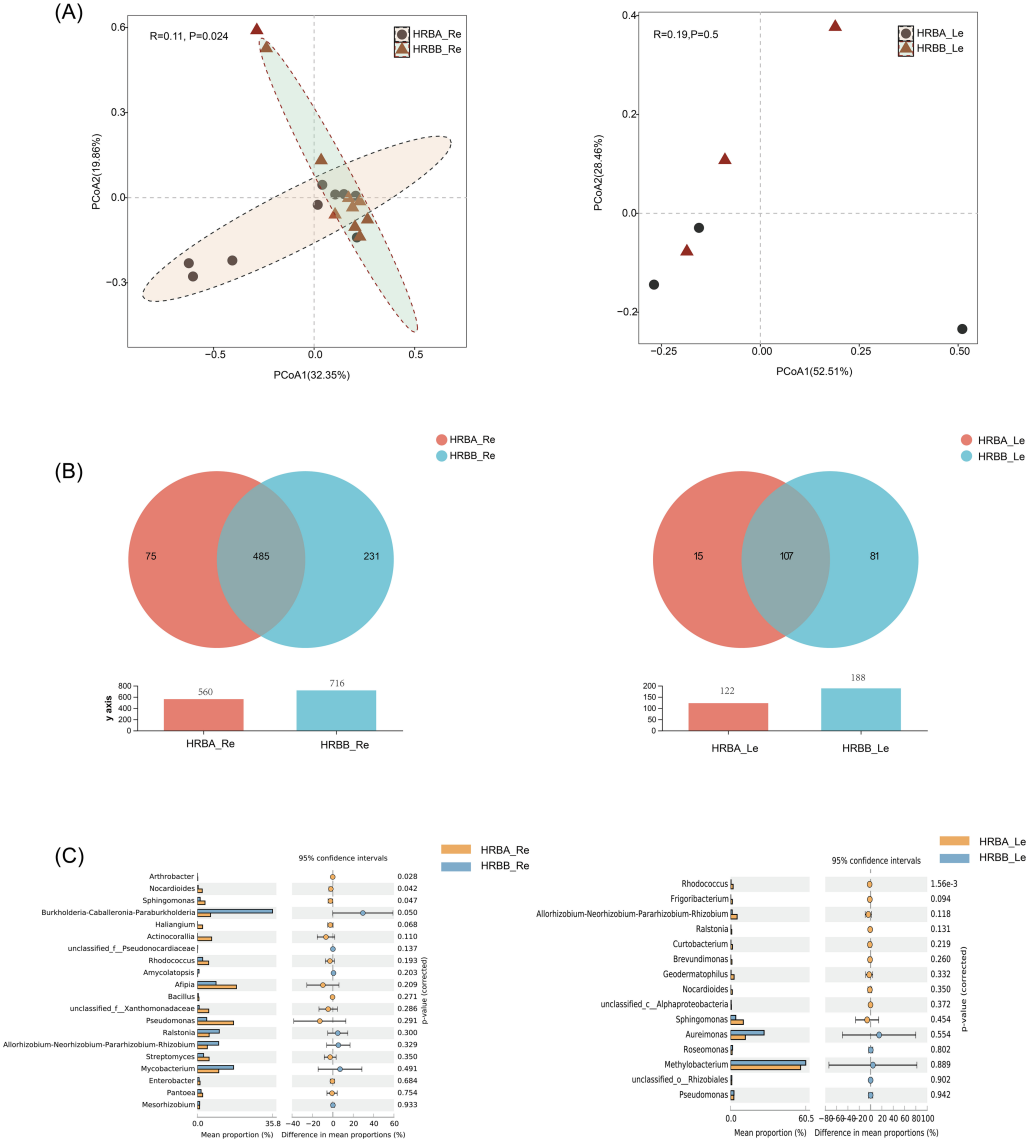
Endophytic bacterial community diversity of HRBA and HRBB. **(A)** Principal component analysis (PCoA) of root and leaf endophytic bacterial communities. **(B)** Venn diagram of endophytic bacteria. **(C)** Differential analysis of endophytic bacterial genera.

To investigate the differences in endophytic microbial communities in different hemp varieties, we first compared the root (Re) and leaf (Le) endophytic bacterial communities of MC (HRBA) and IH (HRBB) cultivated in the same geographic area, around the city of Harbin in Northeast China. The results showed that the diversity of endophytic bacteria in the roots was significantly higher than that in the leaves. Overall, while there was limited difference in bacterial richness and diversity between HRBA_Re and HRBB_Re, principal coordinate analysis (PCoA) revealed significant differences between the communities of HRBA_Re and HRBB_Re (*p* < 0.05). In contrast, while the Shannon and ACE indices showed significant differences between HRBA_Le and HRBB_Le (Supplementary Table S1). The community structure of HRBA_Le and HRBB_Le were very similar to each other (Figure 1A).

Venn diagram analysis indicated that HRBA_Re and HRBB_Re shared 485 OTUs, with 75 OTUs unique to HRBA_Re and 231 OTUs unique to HRBB_Re (Figure 1B). The Wilcoxon rank-sum test revealed significant differences at the genus level, showing that the relative abundances of *Arthrobacter*, *Nocardioides*, and *Sphingomonas* were significantly higher in HRBA_Re compared to HRBB_Re (Figure 1C). For the endophytic bacterial communities in leaves, HRBA_Le and HRBB_Le shared 107 OTUs, with 15 OTUs unique to HRBA_Le and 81 OTUs unique to HRBB_Le (Figure 1B). Additionally, the relative abundance of *Rhodococcus* was significantly higher in HRBA_Le compared to HRBB_Le (Figure 1C). Together, these results revealed both similarities and differences in endophytic bacterial communities between IH and MC in both roots and leaves.

### 3.2 Diversity of endophytic bacteria in industrial hemp and medicinal *Cannabis* across China

We further compared the endophytic bacterial communities between IH and MC cultivars across China. The diversity and richness of endophytic bacteria in the roots and leaves were assessed using Sobs, Shannon, and Ace indices. Overall, the results showed that the diversity and richness of endophytic bacteria were significantly higher in roots than in leaves (Table 2, *p* < 0.05). However, no significant differences were observed between IH_Re and MC_Re. Between IH_Le and MC_Le, the Shannon index was significantly higher in IH_Le than in MC_Le, whereas the ACE index exhibited the opposite trend.

**Table 2:** Diversity indices of endophytic bacteria in industrial hemp (IH) and medicinal *Cannabis* (MC).

Sample	Sobs	Shannon	Ace	Coverage
IH_Re	265.93 ± 13.87^a^	3.03 ± 0.14^a^	402 ± 14.88^a^	0.98
MC_Re	238.19 ± 15.89^a^	3.12 ± 0.15^a^	363.05 ± 18.87^a^	0.98
IH_Le	120.84 ± 10.18^b^	2.76 ± 0.17^ac^	166.63 ± 9.45^b^	0.99
MC_Le	126.57 ± 7.74^b^	2.30 ± 0.13^bc^	222.35 ± 14.13^c^	0.99

Small letters indicate significant differences in endophytic bacteria between industrial hemp and medicinal *Cannabis*. Re, root endophytic; Le, leaf endophytic.

**Figure 2:**
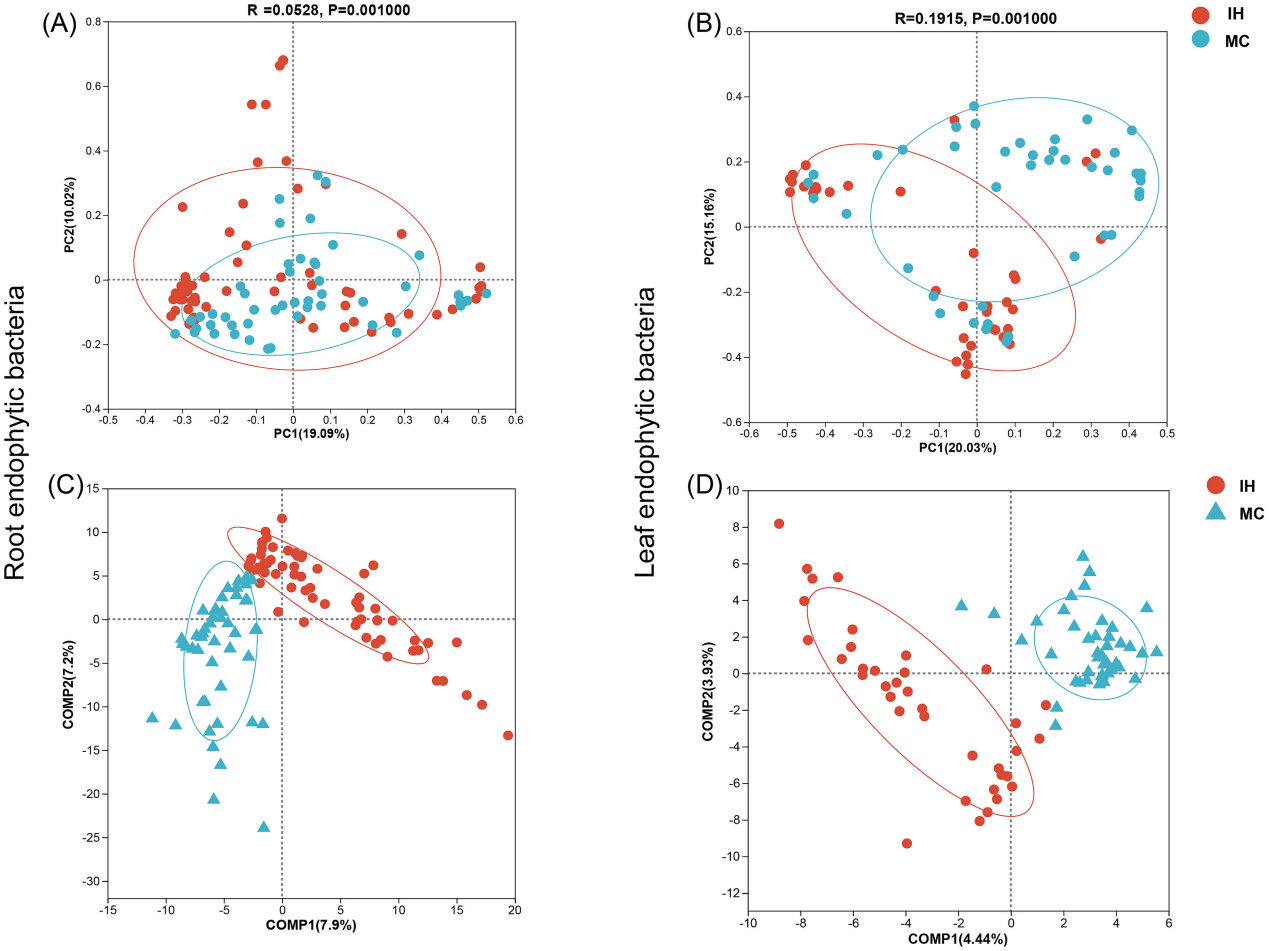
Endophytic bacterial community diversity of industrial hemp and medicinal *Cannabis*. **(A,B)** Principal component analysis (PCoA) of root and leaf endophytic bacterial communities. **(C,D)** Partial least squares discriminant analysis (PLS-DA) of root and leaf endophytic bacterial communities.

Additionally, at the OTU level, partial least squares discriminant analysis (PLS-DA) and PCoA were used to evaluate the similarity of the endophytic bacterial communities. The results showed a significant difference between the endophytic bacterial communities of MC and IH (Figures 2A,B, *p* < 0.01), with clear separation in their clustering patterns (Figures 2C,D). In addition, the differences in endophytic bacteria were more pronounced in the leaves (R = 0.1915) than in the roots (R = 0.0528).

The findings indicate that while the endophytic bacterial communities in the roots of IH and MC cultivars exhibit minimal differences, significant variations were observed in the bacterial communities of the leaves, suggesting cultivar-specific influences on bacterial composition in leaf tissues.

### 3.3 Composition of the endophytic bacteria in industrial hemp and medical *Cannabis*

**Figure 3:**
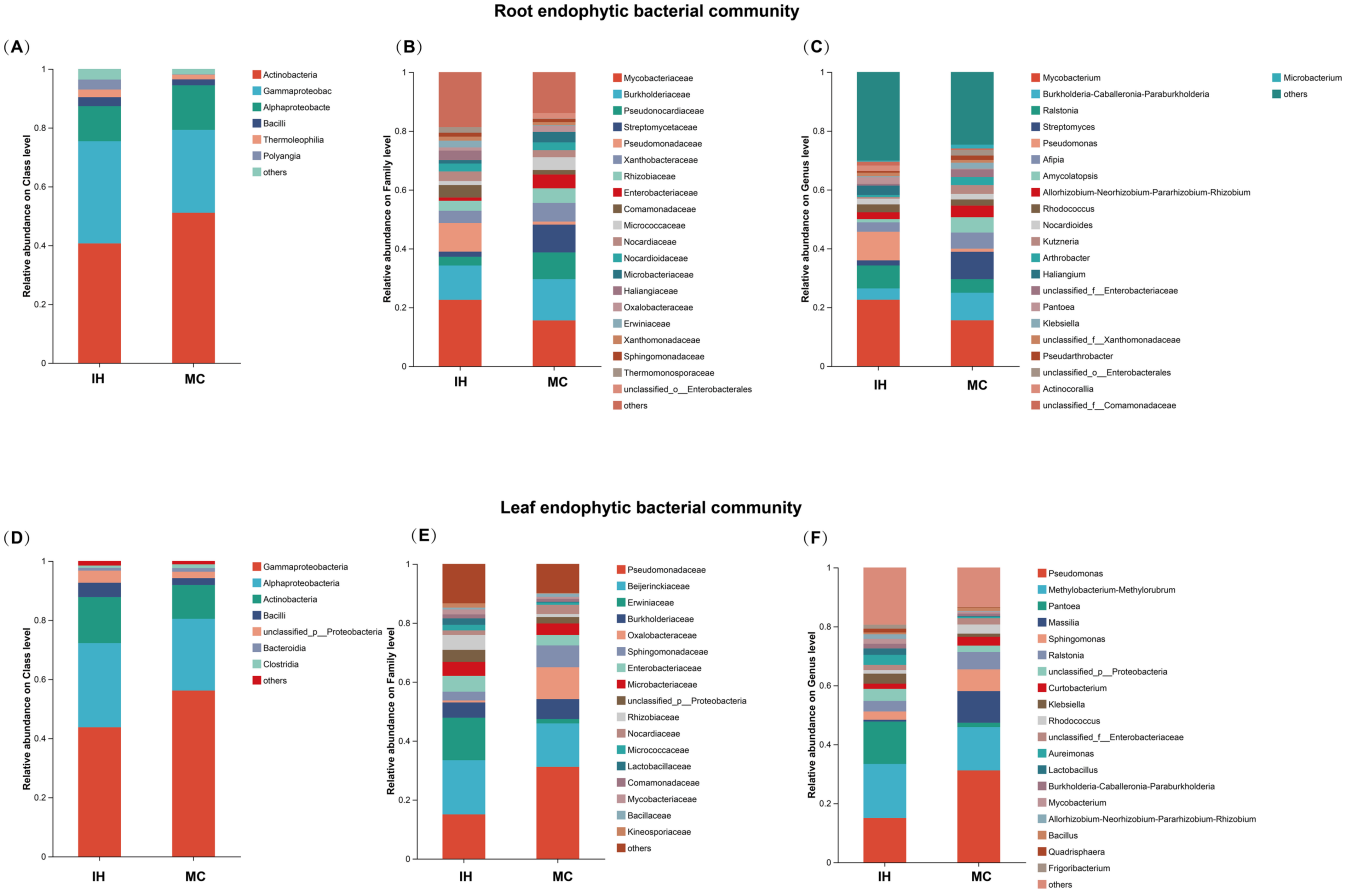
Endophytic bacterial community compositions of industrial hemp and medicinal *Cannabis*. **(A–C)** The relative abundance of dominant root endophytic bacteria. **(D–F)** The relative abundance of dominant leaf endophytic bacteria.

The dominant endophytic bacterial communities in the roots and leaves of industrial hemp (IH) and medicinal *Cannabis* (MC) were analyzed. At the class level (Figures 3A,D), *Actinobacteria*, *Gammaproteobacteria*, *Alphaproteobacteria*, and *Bacilli* are the dominant endophytic bacterial classes (relative abundance > 1%) in both IH and MC. Additionally, IH_Re contains another dominant class, *Polyangia* (3.68%). MC_Le has a higher relative abundance of *Clostridia* (1.30%) and *Bacteroidia* (1.01%).

At the family level (Figure 3B), the dominant families in the root endophytic communities of IH_Re and MC_Re revealed notable differences in their relative abundances. For example, *Mycobacteriaceae* was more abundant in IH_Re (22.57%) than in MC_Re (15.57%), whereas *Burkholderiaceae* exhibited a higher relative abundance in MC_Re (14.05%) compared to IH_Re (11.68%). Furthermore, *Pseudonocardiaceae* (IH: 2.97%, MC: 9.08%) and *Streptomycetaceae* (IH: 1.75%, MC: 9.46%) were more prominent in MC_Re, whereas *Pseudomonadaceae* was significantly more abundant in IH_Re (9.74%) than in MC_Re (1.04%). Similarly, the dominant families in the leaf endophytic communities also revealed distinct distribution patterns (Figure 3E). For instance, *Pseudomonadaceae* had a higher relative abundance in MC_Le (31.18%) than in IH_Le (15.04%), whereas *Erwiniaceae* was significantly more abundant in IH_Le (14.41%) compared to MC_Le (1.50%). Moreover, *Oxalobacteraceae* exhibited a higher relative abundance in MC_Le (10.79%) than in IH_Le (0.68%).

At the genus level, IH and MC have 12 and 15 predominant root endophytic bacterial genera, respectively (Figure 3C), with specific relative abundances detailed in Supplementary Table S2. Notable differences in the composition of predominant endophytic bacterial genera are observed between IH and MC. For instance, *Haliangium* and *Actinocorallia* are predominant in IH, while *Burkholderia-Caballeronia-Paraburkholderia*, *Streptomyces*, *Klebsiella*, *Amycolatopsis*, *Arthrobacter*, *Kutzneria*, *Pseudarthrobacter* and *Microbacterium* are dominant in MC.

Additionally, 15 and 10 predominant leaf endophytic bacterial genera were identified in IH and MC (Figure 3F), respectively. The composition of predominant bacterial genera in the leaves also differs markedly, with *Pseudomonas*, *Pantoea*, and *Massilia* being the representative genera (Supplementary Table S2).

The observed differences in bacterial compositions between IH and MC suggest that cultivar plays a significant role in shaping the structure of endophytic bacterial communities in both roots and leaves.

### 3.4 Shared and unique bacteria in industrial hemp and medical *Cannabis*

**Figure 4:**
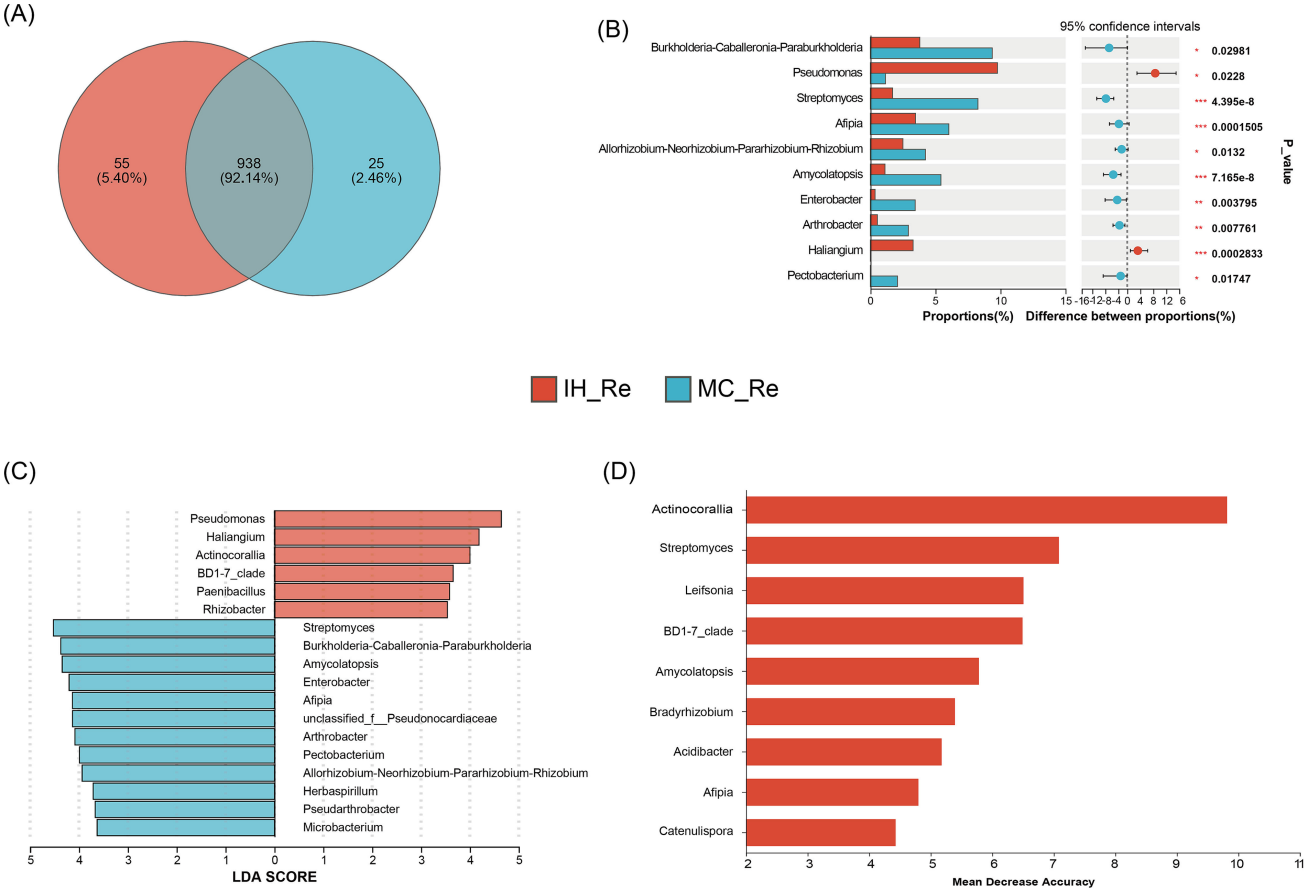
Differential root endophytic bacteria identification between industrial hemp and medical *Cannabis*. **(A)** Venn diagram of root endophytic bacteria. **(B)** Differential analysis of root endophytic bacterial genera. ^*^0.01 < *p* ≤ 0.05; ^**^0.001 < *p* ≤ 0.01; ^***^p ≤ 0.001. **(C)** Graphical summary at the genus in group sample type of biomarkers by LEfSe. **(D)** Significant features identified by random forest.

The comparison of endophytic bacterial communities in the roots and leaves of industrial hemp (IH) and medicinal *Cannabis* (MC) revealed distinct patterns of bacterial diversity and potential biomarkers. The Venn diagram showed that IH_Re and MC_Re shared 938 OTUs, while 55 OTUs were unique to IH_Re and 25 OTUs were unique to MC_Re (Figure 4A). The dominant bacterial genera exhibited a degree of host preference. Analysis of the top 10 dominant endophytic genera using the Wilcoxon rank-sum test revealed that the relative abundances of *Pseudomonas* (*p* < 0.05) and *Haliangium* (*p* < 0.001) were significantly higher in IH_Re than in MC_Re (Figure 4B). Furthermore, linear discriminant analysis effect size (LEfSe, LDA > 3.5) and random forest (RF) were used to identify differential bacteria between industrial hemp and medical *Cannabis* roots. Combining the results of both methods (Figures 4C,D), five biomarker taxa were identified: IH_Re: *Actinocorallia, BD1-7 clade;* MC_Re: *Streptomyces, Amycolatopsis, Afipia.*

**Figure 5:**
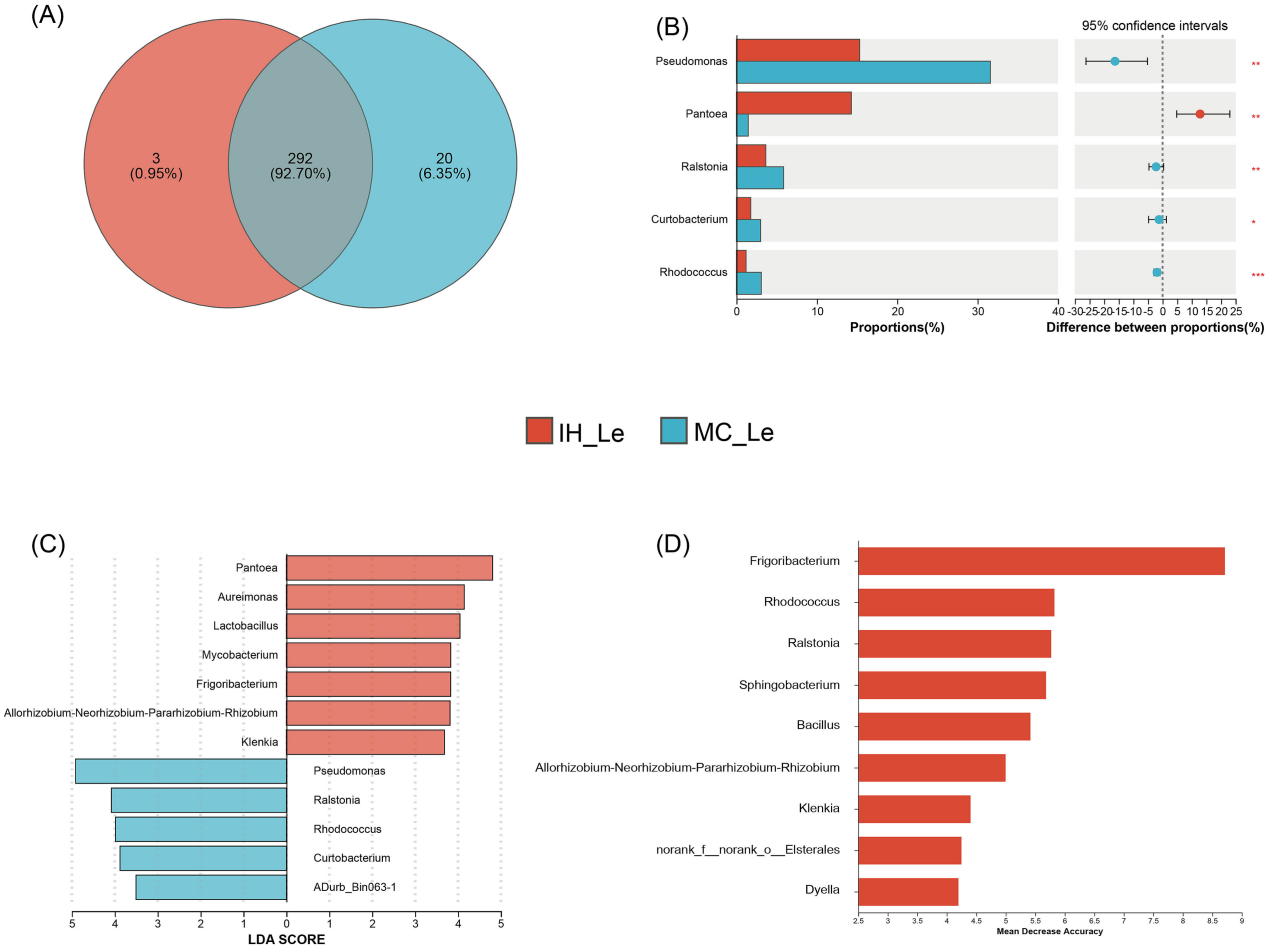
Differential leaf endophytic bacteria of industrial hemp and medical *Cannabis*. **(A)** Venn diagram of leaf endophytic bacteria. **(B)** Differential analysis of leaf endophytic bacterial genera. ^*^0.01 < *p* ≤ 0.05; ^**^0.001 < *p* ≤ 0.01; ^***^p ≤ 0.001. **(C)** Graphical summary at the genus in group sample type of biomarkers by LEfSe. **(D)** Significant features identified by random forest.

In the leaves of industrial hemp and medical *Cannabis*, 292 OTUs were shared, with 3 unique OTUs identified in IH_Le and 20 unique OTUs in MC_Le (Figure 5A). A significance analysis of the top 10 dominant endophytic bacterial genera in the leaves showed that five of the 10 genera exhibited significant differences (Figure 5B). For instance, the relative abundance of *Pantoea* was significantly lower in MC compared to IH, while the relative abundances of the remaining genera were significantly higher in MC than in IH. Additionally, five potential biomarker taxa were identified in the comparison between IH_Le and MC_Le: IH_Le: *Frigoribacterium*, *Allorhizobium-Neorhizobium-Pararhizobium-Rhizobium*, *Klenkia*; MC_Le: *Rhodococcus*, *Ralstonia* (Figures 5C,D).

**Figure 6:**
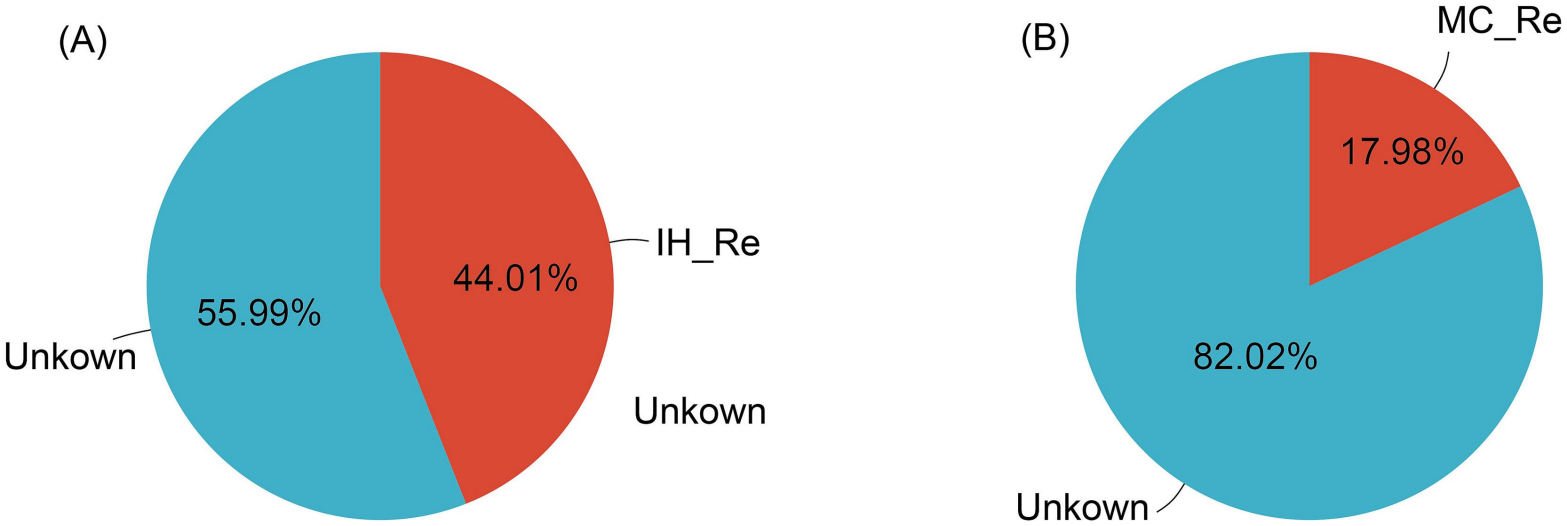
Source tracking analysis of leaf endophytic bacteria. **(A)** Industrial hemp. **(B)** Medicinal *Cannabis*.

Additionally, source tracking analysis was performed on the endophytic bacteria of IH and MC. In IH, 44.01% of the endophytic bacteria in the leaves were shown to be likely originated from the roots, while the remaining 55.99% was classified as unknown. In contrast, only 17.98% of the endophytic bacteria in the leaves of MC were inferred as derived from the roots, with the remaining 82.02% being categorized as unknown (Figure 6).

These results highlight the cultivar-specific characteristics of endophytic bacterial communities, with significant differences in both root and leaf microbiota, suggesting varying ecological features and host-specific influences on bacterial composition.

### 3.5 Function prediction of the endophytic bacteria in industrial hemp and medical *Cannabis*

Functional prediction analyses were conducted using PICRUSt2 and FAPROTAX to compare the functional microbial communities across the root and leaf niches, as well as between the IH and MC cultivars. The Wilcoxon rank-sum test was used to identify significant differences in functional categories. The results revealed distinct metabolic pathways between the two niches, with notable differences in key metabolic processes.

**Figure 7:**
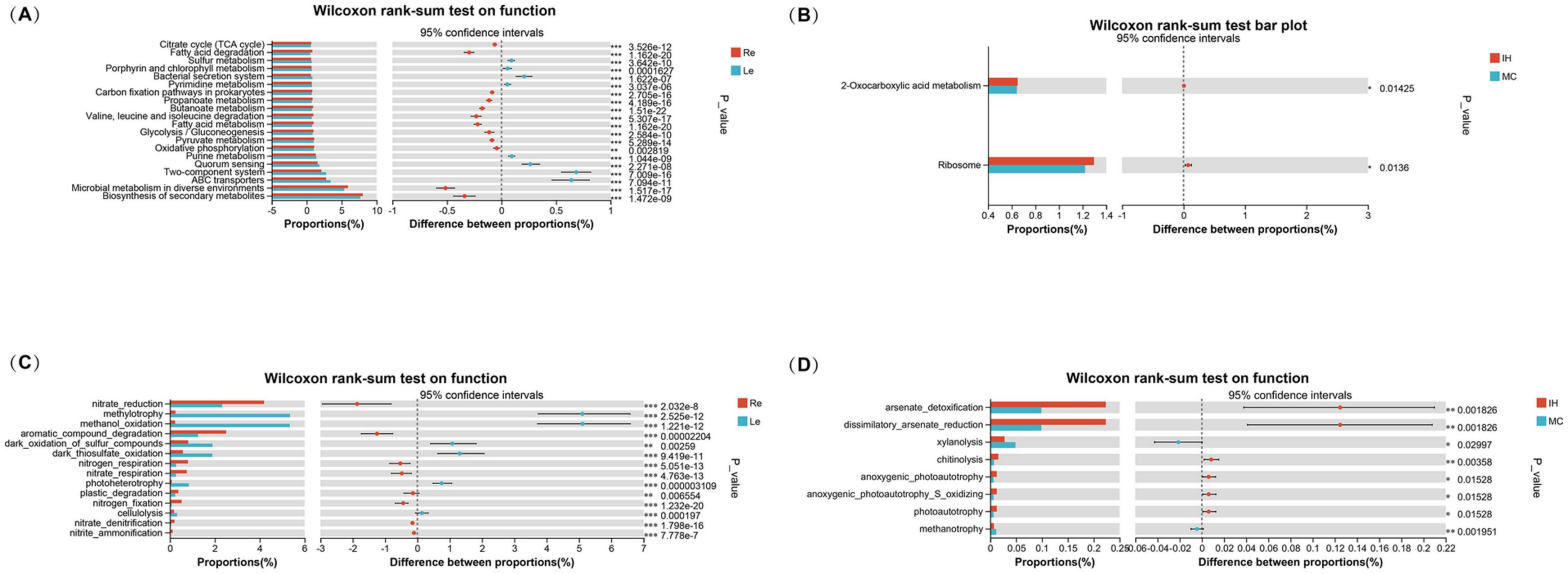
Functional prediction of endophytic bacteria in the roots and leaves of industrial hemp and medicinal *Cannabis*. **(A)** Functional prediction of endophytic bacteria in the roots and leaves based on PICRUSt2. **(B)** Functional prediction of endophytic bacteria in different cultivars based on PICRUSt2. **(C)** Functional prediction of endophytic bacteria in the roots and leaves based on FAPROTAX. **(D)** Functional prediction of endophytic bacteria in different cultivars based on FAPROTAX.

In the Re (Figure 7A), pathways related to the citric acid cycle (TCA cycle), fatty acid degradation, and oxidative phosphorylation were more prevalent, indicating enhanced energy metabolism and oxidative capacity. In contrast, pathways involved in secondary metabolite biosynthesis (such as sulfur metabolism, porphyrin and purine metabolism) were more abundant in the Le, suggesting a higher abundance of microbial communities associated with environmental adaptability and secondary metabolite production.

When comparing the IH and MC cultivars, significant differences were observed in only two functional categories: ribosome function and 2-oxocarboxylic acid metabolism among the top 30 functions (Figure 7B).

FAPROTAX-based predictions further examined the sulfur, nitrogen, hydrogen, and carbon cycles. Functional differences were observed between the ecological niches (Figure 7C). Eight plant growth-related functions, such as nitrate reduction, aromatic compound degradation, nitrogen respiration, nitrate respiration, plastic degradation, nitrogen fixation, nitrate denitrification, and nitrite ammonification, were significantly enriched in the roots. In contrast, the leaves were enriched in functions related to methylotrophy, methanol oxidation, photoheterotrophy, dark thiosulfate oxidation, dark sulfur compound oxidation, and cellulolysis.

Additional revealing eight significant functional differences between the cultivars. IH was enriched in microbial communities associated with arsenate detoxification and dissimilatory arsenate reduction. Additionally, chitinolysis, anoxygenic photoautotrophy, anoxygenic_photoautotrophy_S_oxidizing, and photoautotrophy were significantly more abundant in IH compared to MC. Conversely, xylanolysis and methanotrophy were significantly enriched in MC (Figure 7D).

In summary, the result highlights the complex and dynamic nature of plant-associated microbiomes, with functional differences driven by both plant genotype and ecological niches. Understanding these functional pathways can provide insights into how microbial communities contribute to plant growth, stress responses, and overall plant health.

## 4 Discussion

### 4.1 Factors shaping the *Cannabis* endophytic bacterial composition

The plant microbiome is shaped by multiple biotic and abiotic factors, including soil properties (Sokol et al., 2022), plant cultivars (Matsumoto et al., 2021), environmental conditions (Zhou et al., 2024), and root exudates (Sasse et al., 2018). Soil microbiomes serve as the primary reservoir from which plants recruit their endophytic and rhizosphere-associated microorganisms (Berendsen et al., 2012). Differences in bacterial communities between Industrial hemp (IH) and Medical *Cannabis* (MC) may partially stem from the distinct soil environment in which these plants were cultivated. Soil properties such as Ph (Zhang et al., 2025), nutrient availability (Yan et al., 2025) and organic matter content (Yan et al., 2023) can influence microbial community composition and functional potential, thereby affecting the microbial taxa that ultimately colonize plant tissues (Philippot et al., 2024).

Plant cultivar plays a critical role in microbial selection, particularly in shaping root associated microbiomes. Previous studies have demonstrated that different cultivars can selectively enrich specific microbial taxa due to variations in root exudates (Yue et al., 2023) and plant immune responses (Wang Y. Y. et al., 2022). The differences observed in the bacteria of IH and MC grown in the same region suggest that host genotype influences microbial recruitment beyond the soil microbiome’s initial composition.

### 4.2 Soil influence on bacterial community composition

Our findings indicate that differences in bacterial community composition between IH and MC may, in part, be driven by the distinct soil microbiomes associated with their respective cultivation environments. The variation in dominant bacterial families between IH_Re and MC_Re suggests that microbial recruitment from the soil is a major factor shaping the endophytic microbiome. Soil properties can exert selective pressures on microbial populations, affecting their colonization potential in plant tissues (Duan et al., 2023; Su et al., 2023; Wang et al., 2023).

While the direct impact of soil on leaf endophytic microbiomes is less pronounced, source tracking analysis revealed that a proportion of the endophytic bacteria in leaves originated from the roots, indicating a potential indirect influence. The significantly higher proportion of root-derived bacteria in IH_Le compared to MC_Le suggests that industrial hemp may facilitate more efficient microbial transfer from roots to leaves. This difference could be attributed to host-specific factors such as xylem microbiome transport (Hamaoka et al., 2022), phyllosphere conditions, or systemic signaling mechanisms that influence microbial colonization (Copeland et al., 2015). Future studies incorporating soil physicochemical analysis, metagenomic sequencing, and microbial tracking at multiple plant compartments would provide further insights into the extent of soil influence on the *Cannabis* microbiome, including its indirect effects on aboveground tissues.

### 4.3 Cultivar—dependent bacterial recruitment in a shared environment

In our study, some IH and MC were cultivated in the same geographic region. However, our results revealed significant differences in their endophytic bacterial communities, particularly in the roots. This suggests that host genetic factors play a crucial role in shaping microbial recruitment beyond the influence of the surrounding soil. The observed differences may stem from variations in root exudate composition (Pang et al., 2021), plant immune responses (Castrillo et al., 2017), and physiological traits (Carrión et al., 2019) unique to each cultivar. Root exudates serve as key mediators in microbiome assembly, selectively attracting or repelling specific bacterial taxa (Ji et al., 2023). Previous studies have demonstrated that plant genotypes can influence the quantity and composition of exuded organic compounds, thereby shaping distinct microbial communities even when plants share the same soil (He et al., 2024; Zhang et al., 2019).

In our study, HRBA_Re and HRBB_Re exhibited significant differences in their bacterial communities despite comparable levels of alpha diversity. The enrichment of genera such as *Arthrobacter*, *Nocardioides*, and *Sphingomonas* in HRBA_Re suggests that IH may favor the recruitment of bacteria with potential roles in stress tolerance, biodegradation, or nutrient cycling. Conversely, the significant differentiation in OTUs between HRBA_Re and HRBB_Re indicates that MC recruits a distinct set of bacterial, possibly due to differences in root physiology or interactions with soil microbes.

Interestingly, the leaf microbiota of IH and MC were more similar in composition, with fewer significant differences in dominant taxa. This suggests that while roots play a primary role in shaping the initial endophytic community through direct interactions with the soil, the microbial assembly in leaves may be influenced by factors such as phyllosphere environmental conditions or systemic plant signaling, which could lead to greater homogenization across cultivars. The significantly higher abundance of *Rhodococcus* in HRBA_Le may reflect differences in host physiology or specific plant-microbe interactions that favor certain taxa in the phyllosphere.

Overall, these findings highlight the complex interplay between plant genetics and environmental factors in microbiome assembly. While soil provides a microbial reservoir, host-specific factors ultimately exert selective pressures that shape the final composition of the endophytic community.

### 4.4 Functional adaptations of endophytic bacterial communities in industrial hemp and medicinal *Cannabis*

The functional adaptations of endophytic bacterial communities in industrial hemp (IH) and medicinal *Cannabis* (MC) are shaped by both plant genotype (Hawkes et al., 2021), plant physiology and niche factors (Van Bel et al., 2021). The observed functional differences between IH and MC reflect the divergent ecological niches these cultivars occupy and their distinct physiological roles. Industrial hemp, generally cultivated for its fiber and seeds, often grows in a wider variety of environments, including soils with higher stress levels (e.g., heavy metal contamination or nutrient-poor conditions). This may explain the enrichment of microbial pathways related to arsenate detoxification and dissimilatory arsenate reduction in IH. Such pathways are known to be crucial for the survival of microbes in contaminated soils, suggesting that IH-associated microbiota play a critical role in mitigating environmental stressors. Studies have shown that plants growing in polluted environments often harbor microbiomes with functional traits that enhance stress tolerance (Singh et al., 2023) and pollutant degradation (Tian et al., 2024). In contrast, MC, known for its high resin content and therapeutic properties, shows a functional enrichment in carbon metabolism pathways such as xylanolysis and methanotrophy, which may be linked to the production of bioactive secondary metabolites like THC and CBD. This suggests that MC’s microbiome may support the plant’s metabolic needs for secondary metabolite synthesis, which is vital for its medicinal value. This functional divergence between IH and MC underlines the adaptive role of microbial communities in supporting plant health and productivity in different growing conditions.

The functional differences observed between the root and leaf niches further emphasize that ecological factors drive microbial community specialization. The root was notably enriched in nitrogen-related functions such as nitrate reduction, nitrogen fixation, and nitrate denitrification, all of which are essential for nutrient acquisition and establishing beneficial plant-microbe symbioses in the root zone. These findings align with studies highlighting the role of root-associated microbiota in nitrogen cycling, critical for plant growth and productivity (Pang and Xu, 2024). Conversely, the leaf exhibited a higher abundance of pathways involved in secondary metabolite biosynthesis, including methylotrophy and methanol oxidation, which are linked to the plant’s defense mechanisms and adaptation to oxidative stress (Liu et al., 2023). This suggests that while root microbiota primarily facilitate nutrient cycling and plant growth, leaf microbiota are more involved in environmental stress response, pathogen defense, and plant immunity regulation. These results emphasize the dual roles of microbial communities in plant growth and defense, and highlight the importance of both plant genotype and ecological niches in shaping microbiome functionality.

### 4.5 Limitations of functional predictions from 16S rRNA data

Although 16S rRNA gene sequencing is a valuable tool for characterizing microbial community structure, its application in functional predictions has several limitations (Curry et al., 2022). 16S data primarily provide taxonomic information, and functional predictions are based on known genome databases. However, 16S rRNA genes are highly conserved across species, limiting their ability to capture functional diversity within microbial genomes (Pan et al., 2023). This limitation can lead to inaccurate or incomplete functional predictions, particularly for species with limited genomic data. Additionally, 16S data do not account for non-coding regions or functional genes critical to microbial metabolism and ecological interactions, which are essential for a comprehensive understanding of microbial roles.

Moreover, functional predictions derived from 16S rRNA data are constrained by the completeness of current databases and the challenge of capturing dynamic, environment-specific functions. Microbial community functions are influenced not only by taxonomy but also by factors such as environmental conditions, gene expression, and plant genotype. Therefore, while 16S data can reveal microbial community composition, it often fails to provide a complete picture of functional activity, particularly in specific ecological niches. To overcome these limitations, integrating other high-throughput omics techniques, such as metagenomics or transcriptomics, is essential for generating more accurate and comprehensive functional profiles.

## 5 Conclusion

This study enhances our understanding of plant-microbe interactions by identifying distinct microbial signatures in industrial hemp (IH) and medicinal *Cannabis* (MC), highlighting their ecological and functional implications. Significant differences were observed between the root and leaf microbiomes, emphasizing the crucial roles of both plant genotype and ecological niche in shaping microbial communities. In the roots, *Actinocorallia* and the *BD1-7 clade* were significantly enriched in IH, while MC roots had a higher abundance of *Streptomyces*, *Amycolatopsis*, and *Afipia*. In the leaves, key biomarker taxa such as *Allorhizobium-Neorhizobium-Pararhizobium-Rhizobium*, *Frigoribacterium*, *Klenkia* were enriched in IH, whereas *Rhodococcus* and *Ralstonia* were more abundant in MC. Functional prediction analysis revealed significant differences in metabolic pathways related to nitrogen metabolism, secondary metabolite biosynthesis, and stress tolerance between IH and MC. These findings suggest that microbes in roots play a key role in nitrogen cycling and stress management, while leaves bacteria likely influence the production of bioactive secondary metabolites crucial for the plant’s medicinal properties.

These findings underscore the importance of both plant genotype and ecological niche in shaping microbial communities. While plant genotype has long been recognized as a key driver of microbiome structure, this study demonstrates that environmental factors—such as soil type, nutrient availability, and ecological niche (root vs. leaf)—are equally critical in determining microbial community composition and function. This research provides a more comprehensive understanding of plant-microbe interactions, highlighting how microbial communities in different niches contribute to nutrient cycling, stress tolerance, and secondary metabolite production. The future studies integrating metagenomics and metabolomics will be valuable in further elucidating the molecular mechanisms underlying these plant-microbiome interactions.

## Data Availability

The datasets presented in this study can be found in online repositories. The names of the repository/repositories and accession number(s) can be found at: https://www.ncbi.nlm.nih.gov/, PRJNA1169634 and https://www.ncbi.nlm.nih.gov/, PRJNA1172293.

## Footnotes

^1^ https://cloud.majorbio.com

## Supplementary material

SUPPLEMENTARY FIGURE S1 The rarefaction curve of industrial hemp (IH) and medicinal *Cannabis* (MC).
