# Supplementary figures and images for: DNA metabarcode analyses reveal similarities and differences in plant microbiomes of industrial hemp and medicinal Cannabis in China
Source: Front Microbiol. 2025 Apr 15;16:1524703. doi: 10.3389/fmicb.2025.1524703 (PMC12037489; doi:10.3389/fmicb.2025.1524703)

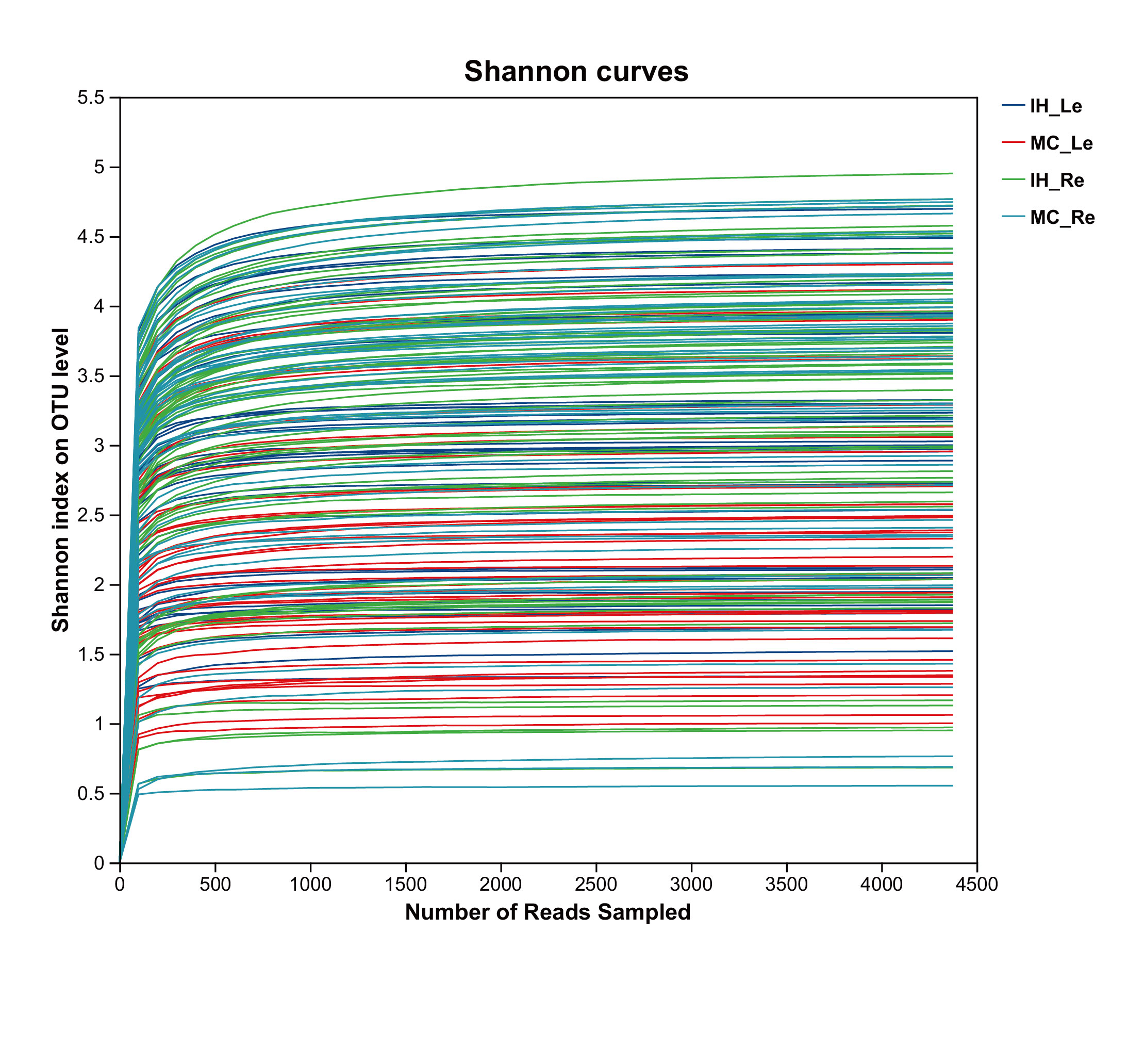

Supplement: SUPPLEMENTARY FIGURE S1 — The rarefaction curve of industrial hemp (IH) and medicinal Cannabis (MC). [file Image_1.jpeg]
